# Expression of LAG-3, TIM-3, and VISTA on Tumor-Infiltrating Lymphocytes in Advanced Laryngeal Squamous Cell Carcinoma and Their Association with CD8+ TIL Density

**DOI:** 10.3390/biomedicines14071587

**Published:** 2026-07-15

**Authors:** Erdoğan Özgür, Ayça Tan, Özlem Yersal, Görkem Eskiizmir, Esin Oktay

**Affiliations:** 1 Department of Otorhinolaryngology–Head and Neck Surgery, Faculty of Medicine, Dokuz Eylül University, İzmir 35340, Türkiye; 2 Department of Medical Pathology, Faculty of Medicine, Manisa Celal Bayar University, Manisa 45030, Türkiye; draycatan@gmail.com; 3 Private Practice, Eskişehir 26180, Türkiye; yersal1978@yahoo.com; 4 Department of Otorhinolaryngology–Head and Neck Surgery, Faculty of Medicine, Manisa Celal Bayar University, Manisa 45030, Türkiye; gorkemeskiizmir@yahoo.com; 5 Department of Medical Oncology, Faculty of Medicine, Aydın Adnan Menderes University, Aydın 09100, Türkiye; esinct@gmail.com

**Keywords:** laryngeal squamous cell carcinoma, LAG-3, TIM-3, VISTA, CD8-positive T-lymphocytes, tumor-infiltrating lymphocytes, immune checkpoints, prognosis

## Abstract

**Background:** Alternative immune checkpoints such as lymphocyte-activation gene 3 (LAG-3), T-cell immunoglobulin and mucin-domain-containing-3 (TIM-3), and V-domain Ig suppressor of T-cell activation (VISTA) have emerged as potential modulators of tumor immune escape. However, their expression patterns and prognostic significance in advanced laryngeal squamous cell carcinoma (LSCC) remain insufficiently characterized. This study aimed to evaluate LAG-3, TIM-3, and VISTA expression on tumor-infiltrating lymphocytes (TILs), examine their association with CD8+ TIL density and clinicopathological features, and determine their impact on survival outcomes. **Methods:** In this retrospective observational cohort study, 132 patients who underwent total or partial laryngectomy for stage III–IV LSCC were included. Tissue microarrays were constructed using three 2 mm cores per case. Immunohistochemical expression of LAG-3, TIM-3, VISTA, and CD8 was assessed exclusively on TILs. Survival outcomes were analyzed using Kaplan–Meier and Cox proportional hazards models. **Results:** LAG-3, TIM-3, and VISTA positivity rates were 26.5%, 51.5%, and 53.8%, respectively. High CD8+ TIL density was observed in 69.7% of cases. Significant positive correlations were identified among checkpoint markers (all *p* < 0.05); VISTA positivity did not significantly correlate with CD8 infiltration (*r* = 0.149, *p* = 0.088). LAG-3 positivity was associated with lower thyroid cartilage invasion (*p* = 0.006). Kaplan–Meier analysis demonstrated no significant differences in overall survival (OS) or disease-free survival (DFS) according to checkpoint expression or CD8 status. In multivariable analysis, extranodal extension (HR = 2.719, *p* = 0.005) and thyroid cartilage invasion (HR = 1.970, *p* = 0.043) were independent predictors of worse OS. CD8 negativity showed a trend toward adverse OS (HR = 1.825, *p* = 0.084). None of the immune markers independently predicted DFS. **Conclusions:** In advanced surgically treated LSCC, LAG-3, TIM-3, and VISTA expression correlate with CD8+ TIL density but do not independently predict survival outcomes. These findings suggest that alternative immune checkpoint expression reflects immune engagement rather than intrinsic tumor aggressiveness and may hold greater predictive than prognostic relevance in LSCC.

## 1. Introduction

Laryngeal cancer represents the second most common malignancy among head and neck cancers and remains a significant cause of morbidity and mortality worldwide. According to Global Cancer Observatory (GLOBOCAN) data, approximately 189,000 new cases are diagnosed annually [1]. Despite advances in surgical techniques, radiotherapy, and systemic therapies, survival outcomes have improved only modestly over the past decades, and overall mortality rates remain substantial. In addition to oncologic control, treatment of laryngeal cancer presents unique challenges due to the complex three-dimensional anatomy of the larynx and its critical functions in phonation, respiration, and airway protection [2]. Standard treatments frequently result in significant functional impairment, including voice loss, dysphagia, and psychosocial distress [3]. These limitations highlight the need for more targeted and biologically informed therapeutic strategies.

The tumor microenvironment (TME) has emerged as a central determinant of tumor progression, immune escape, and treatment resistance. The TME consists of a dynamic interaction between malignant cells, stromal components, immune infiltrates, and extracellular matrix elements. A fundamental hallmark of cancer progression is the ability of tumor cells to evade immune surveillance, a process mediated through multiple mechanisms including the upregulation of immune checkpoint molecules [4]. These inhibitory pathways attenuate T-cell activation and contribute to the establishment of an immunosuppressive microenvironment.

Immune checkpoint inhibitors (ICIs), particularly those targeting programmed death-1 (PD-1) and cytotoxic T-lymphocyte-associated antigen-4 (CTLA-4), have revolutionized the treatment of several malignancies. However, clinical responses remain limited to a subset of patients, and resistance mechanisms are increasingly recognized. This has led to growing interest in alternative immune checkpoint molecules such as lymphocyte-activation gene 3 (LAG-3; official gene symbol: LAG3), T-cell immunoglobulin and mucin-domain-containing-3 (TIM-3; official gene symbol: HAVCR2), and V-domain Ig suppressor of T-cell activation (VISTA; official gene symbol: VSIR). These molecules regulate T-cell exhaustion and immune suppression and are frequently co-expressed in dysfunctional T-cell populations within the TME [5,6].

LAG-3 negatively regulates T-cell activation through binding to major histocompatibility complex (MHC) class II molecules. In head and neck squamous cell carcinoma (HNSCC), particularly in HPV-related oropharyngeal cancers, LAG-3 expression has been shown to correlate with CD8+ T-cell infiltration and may reflect an immunologically active microenvironment [7]. TIM-3, through interaction with ligands such as galectin-9, promotes T-cell exhaustion and reduces cytotoxic anti-tumor responses. Elevated TIM-3 expression has been reported in multiple malignancies and has been associated with immune dysfunction within the TME. VISTA, an inhibitory receptor expressed on both myeloid and T-cell compartments, modulates innate and adaptive immune responses and contributes to immune tolerance within tumors [8,9,10].

Although PD-1/PD-L1 blockade has demonstrated clinical benefit in recurrent or metastatic HNSCC, responses remain heterogeneous, and laryngeal cancer specifically has not shown uniformly robust benefit from current immunotherapeutic strategies [11,12]. These observations underscore the need to explore additional immune regulatory pathways that may contribute to tumor immune evasion.

Recent studies have emphasized the potential of combinatorial immune checkpoint inhibition. Dual blockade strategies, such as LAG-3 combined with PD-1 inhibition, have demonstrated improved response rates compared to monotherapy in melanoma [13,14]. Early-phase clinical trials targeting TIM-3 and VISTA further support the therapeutic relevance of these pathways [15]. In fact, the recent FDA approval of the first dual LAG-3/PD-1 inhibitor combination (relatlimab plus nivolumab) highlights the translational significance of these alternative immune checkpoints.

Despite growing evidence in other tumor types, the expression patterns and prognostic significance of LAG-3, TIM-3, and VISTA in laryngeal squamous cell carcinoma remain insufficiently characterized. Furthermore, the interaction between these immune checkpoints and CD8+ tumor-infiltrating lymphocytes (TILs), a key mediator of anti-tumor immunity, has not been systematically evaluated in advanced laryngeal cancer.

The present study aimed to investigate the expression of LAG-3, TIM-3, and VISTA in advanced laryngeal squamous cell carcinoma, to evaluate their association with CD8+ TIL density and clinicopathological parameters, and to determine their prognostic impact on overall and disease-free survival. A better understanding of these immune regulatory pathways may contribute to the development of more refined immunotherapeutic strategies tailored to laryngeal cancer.

## 2. Materials and Methods

### 2.1. Ethical Approval and Funding

This retrospective observational cohort study was approved by the Non-Interventional Clinical Research Ethics Committee of Aydın Adnan Menderes University Faculty of Medicine (Protocol No: 2021/81; Approval Dates: 10 June 2021/24 and 8 September 2022/11). The study was conducted in accordance with the Declaration of Helsinki. The study was supported by the Scientific Research Projects Unit of Aydın Adnan Menderes University (Project No: TPF-23013)

### 2.2. Study Design and Patient Population

This retrospective observational cohort study included 132 patients who underwent surgical treatment (total or partial laryngectomy) for advanced-stage (Stage III–IV) laryngeal squamous cell carcinoma between January 2010 and December 2020 at Manisa Celal Bayar University Faculty of Medicine Hospital. The surgical approach (total vs. partial laryngectomy) was determined according to tumor extent and institutional practice during the study period. Clinical and pathological data were retrieved from institutional records and included: age and sex, T and N stage, tumor differentiation grade, perineural invasion, lymphovascular invasion, cartilage invasion, lymph node metastasis, extranodal extension, recurrence status, overall survival (OS), and disease-free survival (DFS). Tumors were staged according to the AJCC staging system applicable at the time of diagnosis.

### 2.3. Tissue Microarray (TMA) Construction

Formalin-fixed, paraffin-embedded laryngectomy specimens were retrieved from pathology archives. Hematoxylin–eosin-stained slides were re-evaluated, and representative tumor areas containing both viable tumor tissue and tumor-infiltrating lymphocyte (TIL)-rich regions were selected. From each case, three 2 mm diameter cores were obtained and assembled into tissue microarray (TMA) blocks. A total of eight TMA blocks were constructed. Sections of 4–5 μm thickness were cut from TMA blocks, mounted on positively charged electrostatic slides, and incubated at 58 °C for 4 h. Case-specific barcode labels were applied prior to staining.

### 2.4. Immunohistochemistry

Immunohistochemical staining was performed using a fully automated staining platform (Benchmark Ultra automated IHC/ISH slide staining system, Ventana Medical Systems, Tucson, AZ, USA). The following reagents were used according to the manufacturer’s protocol: UltraView Universal DAB Detection Kit, EZ Prep solution, Reaction Buffer Concentrate, Endogenous Biotin Blocking Kit, Hematoxylin, and Blue Reagent. The following primary antibodies were used: anti-TIM-3 (Cell Signaling Technology, Danvers, MA, USA, clone D5D5R, 1:200), anti-LAG-3 (Cell Signaling Technology, clone CD223, 1:400), anti-VISTA (Cell Signaling Technology, clone D5L5T, 1:400), and anti-CD8 (Cell Marque, clone SP16, 1:50).

### 2.5. Evaluation of Immune Checkpoint Expression

All immunohistochemical evaluations were performed by two experienced pathologists who were blinded to clinical data. Discordant cases were reviewed jointly to reach consensus.

**LAG-3 Evaluation:** LAG-3 expression was assessed on tumor-infiltrating lymphocytes (TILs). In five randomly selected high-power fields (×100 magnification), 100 TILs were counted per field. LAG-3 expression was categorized as: <1% positive TILs → negative and ≥1% positive TILs → positive. This binary cut-off was selected based on previously published literature in head and neck and other solid tumors, where a 1% threshold on TILs has been widely applied for LAG-3 scoring [7,16]. In contrast to TIM-3 and VISTA, for which a semi-quantitative immunoreactivity score incorporating both staining percentage and intensity was used to capture continuous expression gradients, a binary approach was considered sufficient for LAG-3 given its lower overall positivity rate and the precedent established in the existing literature. This 1% threshold is consistent with the validated LAG-3 immunohistochemistry assay used in the RELATIVITY-047 trial of relatlimab plus nivolumab in melanoma, in which LAG-3 positivity was likewise defined using a ≥1% immune-cell cut-off [13,17], and has since been adopted in studies of LAG-3 expression across multiple tumor types. Given the markedly lower overall positivity rate of LAG-3 relative to TIM-3 and VISTA in our cohort, a continuous or multi-tiered scoring system was judged unlikely to provide additional discriminatory value and would have reduced statistical power by further subdividing an already small positive subgroup. We did not formally test alternative LAG-3 cut-offs in the present cohort; we acknowledge this as a methodological limitation and have added this point to Section 4.7.

**TIM-3 and VISTA Evaluation:** TIM-3 and VISTA expression were evaluated exclusively on tumor-infiltrating lymphocytes (TILs). For each case, all three TMA cores were examined individually. In each core, five randomly selected microscopic fields were evaluated at ×100 magnification. In each field, 100 TILs were counted. Lymphocytes showing brown membranous and/or cytoplasmic staining were considered positive. The staining percentage for each core was recorded, and the mean percentage across the three cores was calculated for analysis. A semi-quantitative scoring system was applied: Percentage of positive TILs: Score 1: ≤33%, Score 2: >33–≤66%, Score 3: >66%. Staining intensity: Score 1: absent/weak, Score 2: moderate, Score 3: strong. The final immunoreactivity score was calculated as: Final score = percentage score × intensity score. Total score <3 → low expression; Total score ≥3 → high expression [18]. This combined percentage-intensity (H-score-type) approach is widely used for checkpoint molecules whose expression is more continuously distributed across the cohort, including TIM-3 and VISTA in soft-tissue sarcoma and oral squamous cell carcinoma, where VISTA H-score has itself been shown to associate with disease-free survival [19,20]. We selected this semi-quantitative approach for TIM-3 and VISTA, rather than the binary cut-off used for LAG-3, because both markers showed a substantially higher and more continuously distributed positivity rate in pilot review of our TMA cores, such that a single low cut-off would have classified the majority of cases as positive and discarded clinically relevant gradation in staining intensity. We did not perform a formal sensitivity analysis comparing alternative percentage or intensity cut-offs for TIM-3 and VISTA in the present cohort; this represents a methodological limitation that we now acknowledge explicitly in Section 4.7, and we agree that systematic cut-off optimization (e.g., using ROC-based or maximally selected rank statistics methods) would be a valuable direction for future, larger studies.

**Evaluation of CD8+ Tumor-Infiltrating Lymphocytes:** CD8+ T-cell infiltration was assessed in both intratumoral and peritumoral regions at ×100 magnification. CD8+ TIL density was classified as: <50 lymphocytes/mm^2^ → low infiltration; ≥50 lymphocytes/mm^2^ → high infiltration.

### 2.6. Statistical Analysis

Statistical analyses were performed using SPSS version 29.0 (IBM Corp., Armonk, NY, USA). Descriptive statistics were expressed as mean ± standard deviation or frequency and percentage. Associations between categorical variables were analyzed using the chi-square test. Spearman rank correlation analysis was used to evaluate associations between immune marker expression levels and CD8+ TIL density. Overall survival (OS) and disease-free survival (DFS) were estimated using the Kaplan–Meier method and compared with the log-rank test. Multivariate survival analysis was performed using the Cox proportional hazards regression model. Variables with *p* < 0.10 in univariate analysis, along with clinically relevant parameters established in the laryngeal cancer literature (extranodal extension, cartilage invasion, lymph node metastasis), were considered for inclusion in multivariable models. Given the number of events (40 deaths and 17 recurrences), multivariable models were constructed parsimoniously to reduce the risk of overfitting. OS was defined as the interval between the date of surgery and death from any cause. DFS was defined as the interval between the date of surgery and first documented recurrence. A *p*-value < 0.05 was considered statistically significant. Given the number of comparisons performed in the clinicopathological association analysis, a Bonferroni-corrected significance threshold was additionally applied. As exploratory analyses, survival according to checkpoint marker positivity was examined separately within CD8-high and CD8-low strata using the log-rank test, and unsupervised k-means clustering (k = 2, standardized variables) based on LAG-3 status and mean TIM-3/VISTA staining percentages was performed to identify data-driven patient subgroups independent of pre-specified cutoffs; cluster solutions were evaluated using the silhouette coefficient.

## 3. Results

### 3.1. Patient Characteristics

A total of 132 patients were included in the study. The majority were male (*n* = 124, 93.9%), with a mean age of 60.86 ± 9.06 years (range: 41–83). Total laryngectomy was performed in 97 patients (73.5%), while 35 patients (26.5%) underwent partial laryngectomy. Detailed clinicopathological characteristics are summarized in Table 1.

### 3.2. Immune Marker Expression

**Figure 1:**
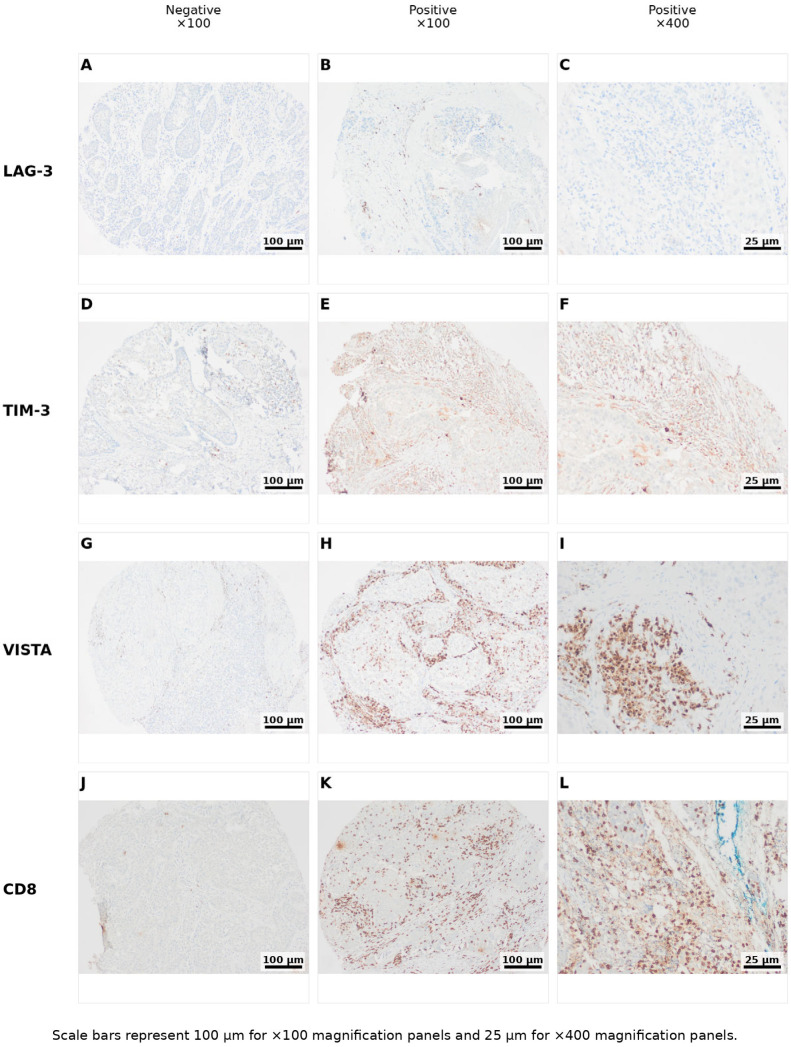
Representative immunohistochemical staining patterns of LAG-3, TIM-3, VISTA, and CD8 in laryngeal squamous cell carcinoma tissue microarray cores. For each marker, a negative core (×100), a positive core (×100), and a higher-magnification view of the positive core (×400) are shown. (**A**–**C**) LAG-3 expression on tumor-infiltrating lymphocytes (TILs): (**A**) negative staining; (**B**) positive staining, scattered membranous positivity; (**C**) ×400 detail showing positive lymphocytes. (**D**–**F**) TIM-3 expression on TILs: (**D**) negative staining; (**E**) positive membranous and cytoplasmic staining; (**F**) ×400 detail showing individual positive cells. (**G**–**I**) VISTA expression on TILs: (**G**) negative staining; (**H**) diffuse positive staining; (**I**) ×400 detail showing clustered positive lymphocytes. (**J**–**L**) CD8+ T-cell infiltration: (**J**) low-density infiltration; (**K**) high-density intratumoral infiltration; (**L**) ×400 detail showing individual CD8+ lymphocytes. Scale bars = 100 μm (×100 panels) and 25 μm (×400 panels).

LAG-3 positivity was observed in 35 patients (26.5%), TIM-3 positivity in 68 (51.5%), and VISTA positivity in 71 (53.8%). High CD8+ tumor-infiltrating lymphocyte (TIL) density (≥50 cells/mm^2^) was present in 92 patients (69.7%). The distribution of immune marker expression levels is detailed in Table 2. Representative immunohistochemical staining patterns are shown in Figure 1.

Spearman rank correlation analysis demonstrated significant positive associations among immune checkpoint markers. VISTA mean expression showed significant correlations with TIM-3 mean expression (*r* = 0.561, *p* < 0.001), and LAG-3 positivity (*r* = 0.350, *p* < 0.001). The correlation between VISTA positivity and CD8 infiltration did not reach statistical significance (*r* = 0.149, *p* = 0.088). Similarly, TIM-3 positivity correlated with LAG-3 positivity (*r* = 0.342, *p* < 0.001) and CD8 infiltration (*r* = 0.317, *p* < 0.001). LAG-3 positivity was also significantly associated with higher CD8 infiltration (*r* = 0.247, *p* = 0.004). In terms of effect size, the VISTA–TIM-3 correlation was of moderate strength, whereas the remaining correlations among checkpoint markers and CD8 infiltration were weak to weak-moderate in magnitude. These findings indicate coordinated expression patterns among immune checkpoint molecules within the tumor microenvironment. Detailed correlation coefficients are presented in Table 3.

### 3.3. Association with Clinicopathological Parameters

LAG-3 positivity was significantly associated with lower rates of thyroid cartilage invasion (*p* = 0.006) and higher CD8 infiltration (*p* = 0.009). No significant associations were observed between LAG-3 expression and other clinicopathological parameters. TIM-3 positivity was not significantly associated with clinicopathological features, except for a significant association with higher CD8 infiltration (*p* < 0.001). VISTA expression did not demonstrate statistically significant associations with clinicopathological parameters in categorical analyses. These associations are summarized in Table 4. After applying a Bonferroni correction for the 30 comparisons tested (corrected significance threshold *p* < 0.00167), only the association between CD8+ TIL density and TIM-3 positivity remained statistically significant (corrected *p* = 0.017). The associations between LAG-3 positivity and thyroid cartilage invasion or CD8+ TIL density did not retain significance after correction (corrected *p* = 0.18 and *p* = 0.26, respectively) and should be interpreted as hypothesis-generating rather than confirmatory.

### 3.4. Survival Analysis

**Figure 2:**
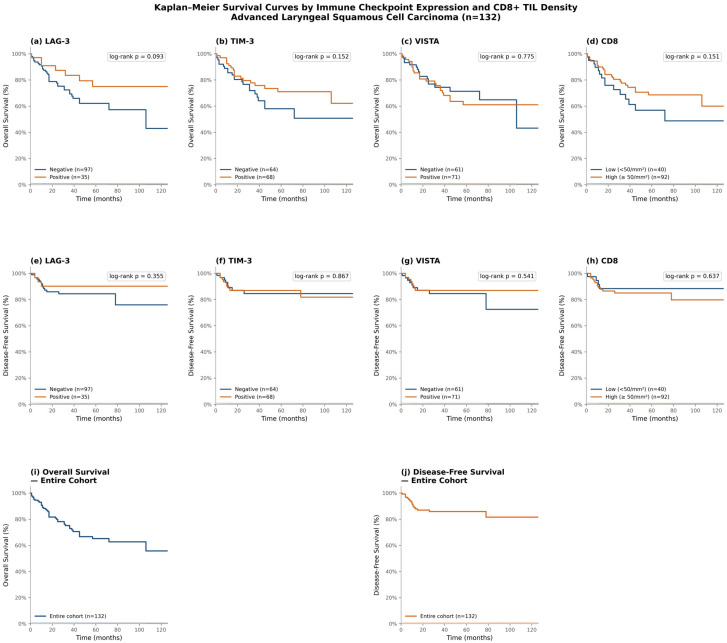
Kaplan–Meier survival curves according to immune checkpoint expression and CD8+ TIL density in advanced laryngeal squamous cell carcinoma (*n* = 132). (**a**–**d**) Overall survival according to LAG-3, TIM-3, VISTA, and CD8 status, respectively. (**e**–**h**) Disease-free survival according to LAG-3, TIM-3, VISTA, and CD8 status, respectively. (**i**) Overall survival for the entire cohort. (**j**) Disease-free survival for the entire cohort. Y-axes show survival probability (%). Shaded areas represent 95% confidence intervals. Log-rank *p* values are shown for each comparison.

The mean follow-up duration was 44.67 ± 33.52 months (range: 1–126 months). During follow-up, 40 patients (30.3%) died and 17 patients (12.9%) developed recurrence. The estimated 2-year OS and DFS rates were 80.0% and 87.0%, respectively. The estimated 5-year OS and DFS rates were 65.2% and 85.9%, respectively. Median OS and DFS were not reached. Kaplan–Meier analysis demonstrated no statistically significant differences in OS or DFS according to LAG-3, TIM-3, VISTA expression status, or CD8+ TIL density (log-rank *p* > 0.05 for all comparisons). Survival curves are presented in Figure 2.

### 3.5. Multivariate Cox Regression Analysis

Overall Survival (OS): Extranodal extension emerged as an independent adverse prognostic factor for OS (HR = 2.719, 95% CI 1.358–5.442, *p* = 0.005). Thyroid cartilage invasion also retained independent significance (HR = 1.970, 95% CI 1.022–3.797, *p* = 0.043). CD8 negativity demonstrated a trend toward worse OS but did not reach statistical significance in the final model (HR = 1.825, 95% CI 0.922–3.611, *p* = 0.084). TIM-3, LAG-3, VISTA expression, cricoid cartilage invasion, and lymph node metastasis were not independently associated with OS. Full univariate and multivariate results are presented in Table 5.

Disease-Free Survival (DFS): In the multivariate model for DFS, none of the immune markers retained independent prognostic significance. Lymph node metastasis demonstrated a borderline association with recurrence risk (HR = 2.730, 95% CI 0.990–7.527, *p* = 0.052), whereas other clinicopathological parameters did not retain statistical significance.

### 3.6. Subgroup and Exploratory Cluster Analysis

To further explore whether the prognostic impact of checkpoint markers differed according to the immunologic context of the tumor, we performed two exploratory analyses. First, survival according to LAG-3, TIM-3, and VISTA positivity was examined separately within CD8-high and CD8-low strata (Table 6). Second, an unsupervised k-means clustering analysis (k = 2, standardized LAG-3 status and mean TIM-3/VISTA staining percentages) was used to identify data-driven patient subgroups based on combined checkpoint expression, independent of pre-specified binary cutoffs.

**Figure 3:**
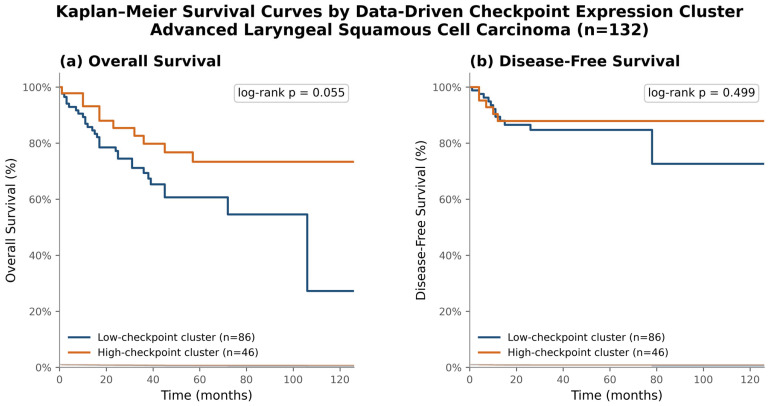
Kaplan–Meier curves according to data-driven checkpoint expression clusters identified by unsupervised k-means clustering (k = 2) based on LAG-3 status and mean TIM-3/VISTA staining percentages. (**a**) Overall survival. (**b**) Disease-free survival. Y-axes show survival probability (%). Shaded areas represent 95% confidence intervals. Log-rank *p* values are shown for each comparison.

In contrast to the binary cutoff-based analyses, unsupervised k-means clustering based on combined checkpoint marker expression identified two distinct patient subgroups: a high-checkpoint cluster (*n* = 46), characterized by LAG-3 positivity in 76.1% of cases and high mean TIM-3 (59.7%) and VISTA (54.9%) staining, and a low-checkpoint cluster (*n* = 86), with no LAG-3 positivity and lower mean TIM-3 (24.7%) and VISTA (24.2%) staining. The high-checkpoint cluster showed a trend toward better overall survival compared with the low-checkpoint cluster (5-year OS approximately 73% vs. 55%; log-rank *p* = 0.055), although this difference did not reach conventional statistical significance. No significant difference in disease-free survival was observed between clusters (log-rank *p* = 0.499). These exploratory findings are presented in Figure 3.

## 4. Discussion

Despite the established clinical benefit of PD-1/PD-L1 blockade in HNSCC [11,21], response rates remain heterogeneous, prompting interest in alternative immune checkpoint molecules as additional mediators of tumor immune escape [5,22].

The present study systematically evaluated CD8+ tumor-infiltrating lymphocyte (TIL) density together with LAG-3, TIM-3, and VISTA expression in advanced laryngeal squamous cell carcinoma (LSCC), and examined their associations with clinicopathological parameters and survival outcomes. To our knowledge, few studies have specifically focused on these alternative immune checkpoints in LSCC in a clinically well-characterized surgical cohort. This represents an important and specific knowledge gap, because available data on LAG-3, TIM-3, and VISTA in HNSCC are derived predominantly from oropharyngeal and oral cavity subsites, which differ substantially from the larynx in etiology (notably HPV-driven carcinogenesis in a relevant proportion of oropharyngeal tumors), anatomical and lymphatic drainage patterns, and baseline immune contexture [7,23]. Extrapolating checkpoint expression data from these HPV-enriched, anatomically distinct subsites to LSCC may not be appropriate, since HPV-positive and HPV-negative HNSCC are increasingly recognized as biologically and immunologically distinct entities with different patterns of T-cell infiltration and checkpoint co-expression. LSCC, which is overwhelmingly HPV-independent and driven primarily by tobacco- and alcohol-related carcinogenesis, therefore warrants dedicated characterization of its immune checkpoint landscape rather than generalization from oropharyngeal data, which the present study aims to address.

### 4.1. CD8+ TIL Density and Prognostic Implications

High CD8+ TIL density was observed in 69.7% of cases in our cohort. In multiple solid tumors, including HNSCC, CD8+ T-cell infiltration has generally been associated with favorable prognosis [24,25]. A recent systematic review confirmed that elevated CD8+ infiltration correlates with improved survival in head and neck cancers [23].

In our series, CD8 negativity demonstrated a trend toward worse overall survival in multivariate analysis, although it did not retain statistical significance in the final model. This finding suggests that CD8 infiltration may contribute to survival outcomes, but its effect is modulated by dominant pathological factors such as extranodal extension and cartilage invasion. The fact that CD8 infiltration did not emerge as an independent prognostic factor in the fully adjusted model highlights the complexity of immune–tumor interactions in LSCC.

Importantly, CD8+ T-cell presence alone may not reflect functional antitumor immunity. T-cell exhaustion, characterized by co-expression of inhibitory checkpoints such as LAG-3 and TIM-3, may limit cytotoxic effectiveness despite abundant infiltration [26]. Thus, quantitative CD8 density must be interpreted within the broader context of immune regulatory signaling.

### 4.2. LAG-3 Expression: Prognostic Ambiguity and Translational Potential

LAG-3 positivity was observed in 26.5% of our cohort, a frequency comparable to that reported in non-small cell lung cancer [16] and colorectal carcinoma [27]. Higher rates have been described in oropharyngeal carcinomas, particularly HPV-associated tumors [7], suggesting that anatomical and viral context may influence checkpoint expression patterns.

The prognostic significance of LAG-3 remains controversial across tumor types. Deng et al. demonstrated that LAG-3 overexpression on TILs correlates with adverse clinicopathological features and confers poor prognosis in HNSCC; however, this study did not provide subsite-specific data for laryngeal cancers [28]. Conversely, studies in gastric cancer and breast carcinoma have reported associations between LAG-3 positivity and improved outcomes [29,30].

In our cohort, LAG-3 positivity was not independently associated with survival; however, LAG-3 negativity was significantly associated with thyroid cartilage invasion. Given that cartilage invasion is a well-established adverse prognostic feature in laryngeal cancer, this association raises the possibility that LAG-3 expression may reflect a more immunologically active microenvironment in tumors with less aggressive local invasion. Nevertheless, this interpretation remains speculative and requires mechanistic validation.

From a therapeutic perspective, LAG-3 holds substantial translational relevance. Preclinical studies have demonstrated that LAG-3 blockade can reinvigorate exhausted T cells, particularly in combination with PD-1 inhibition [14]. The clinical success of dual LAG-3/PD-1 blockade with relatlimab plus nivolumab in melanoma [13] provides compelling proof-of-concept for combinatorial checkpoint targeting. Thus, while LAG-3 did not demonstrate independent prognostic value in LSCC, its role as a potential therapeutic target remains biologically plausible.

### 4.3. TIM-3 Expression and Immune Regulation

TIM-3 overexpression was detected in 51.5% of cases, consistent with frequencies reported in lung, breast, and ovarian cancers [31,32]. The prognostic role of TIM-3 varies widely across tumor types. Some studies associate high TIM-3 expression with poor survival, whereas others report inverse or null associations.

In our series, TIM-3 expression did not correlate with survival outcomes or adverse clinicopathological parameters. However, TIM-3 positivity was significantly associated with higher CD8+ TIL density, supporting the concept that TIM-3 is frequently co-expressed in T-cell-rich microenvironments. Rather than serving as a direct prognostic determinant, TIM-3 may function primarily as an immunoregulatory molecule contributing to T-cell dysfunction within an otherwise inflamed TME. Emerging evidence supports dual TIM-3/PD-1 blockade as a strategy to overcome adaptive resistance to immunotherapy. Therefore, the biological relevance of TIM-3 in LSCC may lie more in its predictive rather than prognostic potential.

### 4.4. VISTA Expression and Methodological Considerations

VISTA positivity was observed in 53.8% of patients in our cohort. This rate is consistent with the frequencies reported across solid malignancies in the literature, and in some tumor types it is even higher. A systematic review and meta-analysis by He et al. [33] demonstrated considerable variability in VISTA expression frequencies across different solid tumors, highlighting the tumor-type-specific nature of VISTA regulation and the difficulty of establishing universal expression benchmarks. Similarly, Martin et al. [34] conducted a comprehensive evaluation of VISTA expression patterns using immunohistochemical approaches across multiple tumor types and emphasized the importance of patient selection strategies based on VISTA status for immune-based anticancer therapies. Taken together, our findings align with this growing body of evidence suggesting that VISTA overexpression is a relevant and recurring feature of the tumor immune microenvironment.

However, the lack of universally accepted cut-off criteria for VISTA—and similarly for TIM-3—continues to complicate meaningful cross-study comparisons. Scoring heterogeneity, arising from differences in antibody clones, staining protocols, and scoring methodologies, represents a significant methodological limitation in the immune checkpoint expression literature [7,35].

In our cohort, VISTA expression did not demonstrate independent prognostic significance in multivariate analysis. Nevertheless, its positive correlation with CD8+ tumor-infiltrating lymphocyte (TIL) density and co-expression with other immune checkpoint molecules suggests a pattern of coordinated immune regulatory activity within the TME. This observation is consistent with prior reports demonstrating that VISTA expression correlates with CD8+ infiltration and may reflect an adaptive immunosuppressive response to cytotoxic T-cell activity [36,37]. VISTA is increasingly recognized as a critical modulator of both innate and adaptive immunity, operating across myeloid and lymphoid compartments to maintain peripheral tolerance and promote immune evasion [9,38].

### 4.5. Integrated Interpretation

Taken together, the findings from the individual marker analyses converge on a coherent biological picture: LAG-3, TIM-3, and VISTA are co-expressed in a coordinated manner and correlate positively with CD8+ TIL density, yet none independently predicts survival. This integrated pattern supports the concept of a shared immunoregulatory landscape within the LSCC tumor microenvironment rather than marker-specific prognostic effects [7,36].

This interpretation was further supported by our exploratory subgroup and clustering analyses. Survival comparisons stratified by CD8+ TIL density did not reveal significant differences for any marker within either stratum, although the CD8-low subgroup was particularly underpowered. Notably, an unsupervised, data-driven clustering approach based on combined checkpoint expression separated patients into a high-checkpoint and a low-checkpoint phenotype that showed a non-significant trend toward better survival in the high-checkpoint group. This pattern is consistent with an inflamed-yet-exhausted phenotype in which coordinated checkpoint upregulation marks a more immunologically engaged, rather than more aggressive, tumor microenvironment. Given the modest sample size, these subgroup and cluster-based findings should be regarded as hypothesis-generating and warrant confirmation in larger, multi-institutional cohorts.

### 4.6. Mechanistic Considerations and Therapeutic Implications

The positive correlations observed between CD8+ TIL density and LAG-3, TIM-3, and VISTA expression are biologically consistent with established mechanisms of adaptive immune resistance. Chronic antigen exposure within an actively infiltrated tumor microenvironment drives progressive T-cell exhaustion, characterized by stepwise upregulation of multiple co-inhibitory receptors on the same CD8+ T-cell populations responsible for antitumor cytotoxicity [5,6]. Experimental models of acquired resistance to PD-1/PD-L1 blockade have shown that TIM-3 is upregulated as a compensatory mechanism on CD8+ T cells and regulatory T cells following PD-1 pathway inhibition, including in human HNSCC tumor-infiltrating lymphocytes specifically, representing one of several adaptive escape mechanisms by which tumors re-establish immunosuppression when a single checkpoint is blocked [39]. LAG-3 and VISTA are similarly co-expressed with PD-1 on exhausted CD8+ T cells and, in the case of VISTA, are also expressed on myeloid and regulatory T-cell populations, broadening their potential immunosuppressive reach beyond the CD8+ compartment itself [9,10]. The coordinated, rather than independent, expression pattern of LAG-3, TIM-3, and VISTA observed in our cohort is therefore consistent with a shared, CD8-driven exhaustion program rather than three biologically unrelated processes.

From a therapeutic perspective, this biology provides a clear rationale for combinatorial rather than sequential checkpoint blockade in LSCC. Because LAG-3 and TIM-3 act through mechanisms distinct from PD-1 and from one another, single-agent blockade of any one pathway may be insufficient to fully reverse T-cell dysfunction, whereas dual blockade has been shown to produce more robust reactivation of exhausted CD8+ T cells than either pathway alone, as demonstrated clinically by the approval of relatlimab plus nivolumab in melanoma [13]. The high baseline co-expression of LAG-3, TIM-3, and VISTA with CD8+ TIL density observed in our LSCC cohort suggests that a relevant subset of patients—particularly those with high CD8+ infiltration but disease progression on PD-1/PD-L1-based regimens—could plausibly benefit from the addition of LAG-3- or TIM-3-targeted agents. We acknowledge that this implication is necessarily speculative in the absence of treatment-outcome data with checkpoint inhibitors in our own cohort, and that prospective biomarker-driven trials in LSCC specifically will be required to test this hypothesis.

Instead, classical histopathological parameters—particularly extranodal extension (ENE) and thyroid cartilage invasion—remained the dominant prognostic determinants in multivariate analysis. ENE has been consistently identified as an independent adverse prognostic factor in LSCC, with ENE-positive patients demonstrating markedly inferior overall survival compared to both node-negative and ENE-negative node-positive cases [40,41]. Similarly, thyroid cartilage infiltration plays a critical role in disease staging and local tumor behavior, and its extent has been shown to carry prognostic implications in locally advanced LSCC [42]. These findings reinforce the notion that immune checkpoint expression reflects the immunological state of the host–tumor interface rather than the intrinsic biological aggressiveness of the tumor [22].

The observed checkpoint–CD8 correlations are consistent with an “inflamed yet exhausted” immune phenotype—a state in which robust cytotoxic T-cell infiltration is paradoxically accompanied by functional impairment mediated through inhibitory receptor upregulation [43,44]. In such a context, immune checkpoint expression alone is insufficient to alter the natural disease course but may identify a subset of tumors that are biologically more amenable to combinatorial immunotherapeutic intervention.

Importantly, lack of independent prognostic significance should not be interpreted as lack of biological relevance. This distinction between prognostic and predictive biomarkers is critical and well-established in oncology [45,46]. While LAG-3, TIM-3, and VISTA did not independently determine survival in this surgically treated cohort, their coordinated association with CD8+ TIL density suggests potential predictive value in the setting of immunotherapeutic modulation. Checkpoint expression appears to reflect the state of host–tumor immune interaction rather than the biological capacity of the tumor to invade or metastasize. Thus, alternative immune checkpoints in LSCC may hold greater therapeutic than prognostic significance, warranting prospective evaluation in the context of ICI-based combination strategies.

### 4.7. Limitations

This study has limitations inherent to its retrospective, single-center design. The sample size, while substantial for a surgically treated LSCC cohort, may limit detection of modest survival effects. Universally standardized cut-off values for TIM-3 and VISTA immunohistochemistry are lacking across the literature, and we did not formally test alternative thresholds in the present cohort; this introduces potential variability when comparing our findings with other studies and represents an important target for future cut-off optimization analyses (e.g., ROC-based or maximally selected rank statistics approaches). Tissue microarray sampling, while practical for high-throughput biomarker assessment, may not fully capture the substantial spatial heterogeneity of immune infiltration described in LSCC and other HNSCC subsites; although three 2 mm cores per case is consistent with published validation studies showing good concordance between triplicate cores and whole-tissue sections for several immunohistochemical markers [47], TMA-based sampling can still underestimate spatially restricted or focal checkpoint expression and cannot be assumed to perform equally well for every biomarker [48]. Of note, the number of observed events (40 deaths and 17 recurrences) was modest relative to the number of variables examined. Although multivariable models were constructed parsimoniously to minimize overfitting, the limited event count—especially for DFS—may have constrained statistical power and contributed to the lack of independent significance of certain immune parameters. Importantly, the absence of statistical significance in these survival analyses should not be equated with an absence of biological relevance: with the limited event numbers available, particularly for DFS, our study was likely underpowered to detect modest but biologically meaningful effects of checkpoint expression on outcome, and the consistent direction of associations across markers (e.g., higher CD8+ TIL density and coordinated checkpoint expression) may still be clinically informative despite not reaching conventional significance thresholds.

## 5. Conclusions

In advanced laryngeal squamous cell carcinoma, LAG-3, TIM-3, and VISTA expression are significantly associated with CD8+ TIL density but do not independently predict overall or disease-free survival. These findings suggest that alternative immune checkpoint expression reflects immune microenvironment activation rather than serving as a direct prognostic biomarker. Future prospective studies incorporating functional immune profiling and combined checkpoint expression analyses—particularly in the context of PD-1/PD-L1 blockade—are warranted. As combination immunotherapy strategies continue to evolve, alternative checkpoints such as LAG-3, TIM-3, and VISTA may assume increasing importance as predictive biomarkers and therapeutic targets in laryngeal cancer.

## Figures and Tables

**Table 1:** Clinicopathological Characteristics of the Study Population (*n* = 132).

Characteristic	*n*	%
**Age (years)**		
Mean ± SD (range)	60.86 ± 9.06 (41–83)	
**Sex**		
Male	124	93.9
Female	8	6.1
**Surgery type**		
Total laryngectomy	97	73.5
Partial laryngectomy	35	26.5
**Tumor localization**		
Supraglottic	36	27.3
Glottic	11	8.3
Subglottic	3	2.3
Supraglottic + Glottic	7	5.3
Glottic + Subglottic	26	19.7
Transglottic	49	37.1
**Tumor differentiation**		
Well	19	14.4
Moderate	92	69.7
Poor	21	15.9
**Pathological T stage**		
T1	5	3.8
T2	17	12.9
T3	45	34.1
T4	65	49.2
**Pathological N stage**		
N0	80	60.6
N1	14	10.6
N2	13	9.8
N3b	25	18.9
**Thyroid cartilage invasion**		
Absent	82	62.1
Present	50	37.9
**Cricoid cartilage invasion**		
Absent	115	87.1
Present	17	12.9
**Lymphovascular invasion**		
Absent	121	91.7
Present	11	8.3
**Perineural invasion**		
Absent	116	87.9
Present	16	12.1
**Surgical margin**		
Negative	95	72.0
Positive	37	28.0
**Lymph node metastasis**		
Absent	80	60.6
Present	52	39.4
**Extranodal extension**		
Absent	96	72.7
Present	26	19.7
**Adjuvant therapy**		
None	33	25.0
RT alone	30	22.7
RT + CT	69	52.3
**Distant metastasis**		
Absent	118	89.4
Present	14	10.6
**Recurrence**		
Absent	115	87.1
Present	17	12.9
**Survival status**		
Alive	92	69.7
Dead	40	30.3
**Follow-up duration (months)**		
Mean ± SD (range)	44.67 ± 33.52 (1–126)	

RT: radiotherapy; CT: chemotherapy. Percentages may not sum to 100% due to rounding or missing values.

**Table 2:** Distribution of Immune Checkpoint Marker and CD8+ TIL Expression.

Marker	Low/Negative *n* (%)	High/Positive *n* (%)	Mean Staining (%) ± SD (All Cases)
**LAG-3**	97 (73.5)	35 (26.5)	2.71 ± 3.49 (positive cases only)
**TIM-3**	64 (48.5)	68 (51.5)	36.90 ± 28.04
**VISTA**	61 (46.2)	71 (53.8)	34.90 ± 24.96
**CD8+ TILs (<50 vs. ≥50 cells/mm^2^)**	40 (30.3)	92 (69.7)	–

LAG-3: mean staining percentage reported for positive cases only (*n* = 35); binary scoring applied. TIM-3 and VISTA: mean staining percentage calculated across all 132 cases. SD: standard deviation; TILs: tumor-infiltrating lymphocytes.

**Table 3:** Spearman Rank Correlation Analysis Between Immune Checkpoint Markers and CD8+ TIL Density.

Variable	VISTA (Mean)	TIM-3 (Mean)	LAG-3 (Positive)	CD8 Infiltration
**VISTA (Mean)**	1	0.561 ***	0.350 ***	0.149
**TIM-3 (Mean)**		1	0.342 ***	0.317 ***
**LAG-3 (Positive)**			1	0.247 **
**CD8 Infiltration**				1

Spearman correlation coefficients (r) are shown. ** *p* < 0.01, *** *p* < 0.001. VISTA and TIM-3 represent mean expression percentages; LAG-3 and CD8 represent binary positivity status.

**Table 4:** Association of Immune Checkpoint Marker Positivity with Clinicopathological Parameters.

Parameter	Category	LAG-3 Pos %	*p*	TIM-3 Pos %	*p*	VISTA Pos %	*p*
**Thyroid cartilage invasion**	Absent	35.4	**0.006 ^†^**	53.7	0.652	53.7	1.000
	Present	12.0		48.0		54.0	
**Cricoid cartilage invasion**	Absent	27.8	0.558	51.3	1.000	53.0	0.853
	Present	17.6		52.9		58.8	
**T stage**	T1–T4	—	0.052	—	0.301	—	0.880
**Perineural invasion**	Absent	28.4	0.235	52.6	0.692	55.2	0.554
	Present	12.5		43.8		43.8	
**Lymphovascular invasion**	Absent	26.4	1.000	52.9	0.355	53.7	1.000
	Present	27.3		36.4		54.5	
**Lymph node metastasis**	Absent	31.4	0.290	52.9	0.734	58.6	0.334
	Present	21.2		48.1		48.1	
**Extranodal extension**	Absent	29.2	0.446	52.1	0.752	53.1	0.847
	Present	19.2		46.2		57.7	
**CD8+ TIL density**	≥50/mm^2^	33.7	**0.009 ^†^**	62.0	**<0.001 ^‡^**	58.7	0.127
	<50/mm^2^	10.0		27.5		42.5	
**Distant metastasis**	Absent	28.0	0.352	51.7	1.000	52.5	0.582
	Present	14.3		50.0		64.3	
**Recurrence**	Absent	27.8	0.558	51.3	1.000	54.8	0.737
	Present	17.6		52.9		47.1	
**Survival status**	Alive	30.4	0.183	54.3	0.425	52.2	0.708
	Dead	17.5		45.0		57.5	

Values represent the percentage of marker-positive cases within each category. *p* values from chi-square or Fisher’s exact test. Bold *p* values: *p* < 0.05. Given the 30 pairwise comparisons performed across the three markers and ten clinicopathological parameters, a Bonferroni-corrected significance threshold of *p* < 0.00167 (0.05/30) was applied. ^†^ Associations significant at the nominal *p* < 0.05 level that did not retain significance after Bonferroni correction (thyroid cartilage invasion–LAG-3, corrected *p* = 0.18; CD8+ TIL density–LAG-3, corrected *p* = 0.26). ^‡^ Association that remained statistically significant after Bonferroni correction (CD8+ TIL density–TIM-3, corrected *p* = 0.017).

**Table 5:** Univariate and Multivariate Cox Regression Analysis for Overall Survival.

Variable	Univariate HR (95% CI)	*p*	Entered into Final Model	Multivariate HR (95% CI)	*p*
LAG-3 positivity	0.500 (0.220–1.140)	0.099	No	—	—
TIM-3 positivity	0.634 (0.338–1.190)	0.156	No	—	—
VISTA positivity	1.098 (0.586–2.058)	0.770	No	—	—
CD8 negativity (ref: high CD8+ TIL)	1.593 (0.838–3.028)	0.155	Yes	1.825(0.922–3.611)	0.084
Extranodal extension	2.693 (1.375–5.276)	**0.004**	Yes	2.719(1.358–5.442)	**0.005**
Thyroid cartilage invasion	2.159 (1.155–4.033)	**0.016**	Yes	1.970 (1.022–3.797)	**0.043**
Cricoid cartilage invasion	1.740 (0.800–3.782)	0.162	No	—	—
Lymph node metastasis	1.737 (0.931–3.239)	0.083	No	—	—
Perineural invasion	1.298 (0.503–3.349)	0.590	No	—	—
Lymphovascular invasion	1.334 (0.410–4.343)	0.632	No	—	—

All ten clinicopathological and immune marker variables were first tested in univariate Cox models. Variables with univariate *p* < 0.10 (extranodal extension, thyroid cartilage invasion, lymph node metastasis), together with CD8+ TIL status as a clinically pre-specified covariate, were considered for the multivariable model; lymph node metastasis was not retained in the final model owing to collinearity with extranodal extension and lack of independent contribution. The final parsimonious model (3 covariates, 40 events) is shown in the Multivariate columns. Bold *p* values: *p* < 0.05. HR: hazard ratio; CI: confidence interval.

**Table 6:** Subgroup Survival Analysis Stratified by CD8+ TIL Density.

CD8 Stratum	Marker	Outcome	Marker-Positive *n* (Events)	Marker-Negative *n* (Events)	Log-Rank *p*
CD8-high	LAG-3	OS	31 (7)	61 (18)	0.364
CD8-high	LAG-3	DFS	31 (5)	61 (15)	0.356
CD8-high	TIM-3	OS	57 (14)	35 (11)	0.277
CD8-high	TIM-3	DFS	57 (12)	35 (8)	0.646
CD8-high	VISTA	OS	54 (15)	38 (10)	0.917
CD8-high	VISTA	DFS	54 (11)	38 (9)	0.632
CD8-low	LAG-3	OS	4 (0)	36 (15)	0.201
CD8-low	LAG-3	DFS	4 (0)	36 (8)	0.384
CD8-low	TIM-3	OS	11 (4)	29 (11)	0.805
CD8-low	TIM-3	DFS	11 (2)	29 (6)	0.761
CD8-low	VISTA	OS	17 (8)	23 (7)	0.240
CD8-low	VISTA	DFS	17 (4)	23 (4)	0.486

Patients were stratified by CD8+ TIL density (high ≥50/mm^2^, *n* = 92; low <50/mm^2^, *n* = 40), and the association between each checkpoint marker and overall survival (OS) or disease-free survival (DFS) was tested separately within each stratum using the log-rank test. No subgroup comparison reached statistical significance, although the analysis was limited by reduced sample size and event numbers within strata, particularly in the CD8-low subgroup (e.g., only 4 LAG-3-positive patients with 0 events).

## Data Availability

The data presented in this study are available from the corresponding author upon reasonable request. The data are not publicly available because they contain information that could compromise the privacy of the research participants.
